# Repetitive Transcranial Magnetic Stimulation Coupled With Visual-Feedback Cycling Exercise Improves Walking Ability and Walking Stability After Stroke: A Randomized Pilot Study

**DOI:** 10.1155/np/8737366

**Published:** 2024-11-26

**Authors:** Yixiu Wang, Xiaoming Chen, Menghuan Wang, Yingying Pan, Shiyi Li, Mengfei He, Feng Lin, Zhongli Jiang

**Affiliations:** ^1^School of Rehabilitation Medicine, Nanjing Medical University, Nanjing, Jiangsu, China; ^2^Department of Rehabilitation Medicine, The First Affiliated Hospital of Nanjing Medical University, Nanjing, Jiangsu, China; ^3^Department of Rehabilitation Medicine, Nanjing Hospital of Chinese Medicine, Nanjing, Jiangsu, China; ^4^Department of Rehabilitation Medicine, Sir Run Run Hospital, Nanjing Medical University, Nanjing, Jiangsu, China

**Keywords:** cycling exercise, lower extremity function, motor evoked potentials, repetitive transcranial magnetic stimulation, stroke rehabilitation

## Abstract

**Background:** Stroke survivors exhibit persistent abnormal gait patterns, particularly in diminished walking ability and stability, limiting mobility and increasing the risk of falling. The purpose of the study was to determine the effects of repetitive transcranial magnetic stimulation (rTMS) coupled with cycling exercise on walking ability and stability in patients with stroke and explore the potential mechanisms underlying motor cortex recovery.

**Methods:** In this double-blinded randomized pilot trial, 32 stroke patients were randomly separated into the real-rTMS group (RG, receiving rTMS during active cycling exercise) and the sham-rTMS group (SG, receiving sham rTMS during active cycling exercise). Participants completed 10 exercise sessions (5 times per week). Lower extremity function was measured using the Fugl-Meyer assessment of lower extremity (FMA-LE), and functional balance ability was measured by the Berg balance scale (BBS). The 2-min walk test (2MWT) and standing balance test were employed to evaluate walking and balance ability. Motor evoked potentials (MEPs) were measured to evaluate cortical excitability. The above assessments were administered at baseline and after the intervention. Additionally, the cycling exercise performance was recorded after the initial and final exercise sessions to evaluate the motor control during exercise.

**Results:** The RG showed significant improvements in lower extremity function (FMA-LE) and functional balance ability (BBS) compared to the SG at postintervention. The walking and balance abilities, as well as the motor asymmetry of cycling exercise, significantly improved in RG. Additionally, participants in RG exhibited a higher elicitation rate of ipsilesional MEPs than that in SG. The improvements in motor asymmetry of cycling exercise in RG were significantly associated with increases in FMA-LE scores and walking ability.

**Conclusion:** The combination of rTMS and cycling exercise effectively improves walking ability and walking stability in patients with stroke, which may be related to the excitability modulation of the motor cortex induced by rTMS.

**Trial Registration**: Clinical Trial Registry identifier: ChiCTR2400079360.

## 1. Introduction

As a common cerebrovascular disease, stroke has been the second leading cause of death worldwide [1]. Research indicates that approximately two-thirds of stroke survivors experience lower extremity motor dysfunction, which severely impacts mobility and the performance of daily life activities [2]. Current stroke rehabilitation provided to patients with lower limb dysfunction primarily focuses on improvements in basic gait parameters, such as better walking speed and further distance [3, 4]. Stroke patients may obtain the ability to walk independently after rehabilitation therapy, but most of them continue to exhibit abnormal gait patterns, which result in low walking efficiency and poor walking stability, heightening the risk of falls [5]. Goal-oriented, active, repetitive movements in walking training are widely used to improve gait by enhancing motor relearning and promoting neuroplasticity [6]. However, declines in motor control and muscle strength are the primary motor deficits leading to abnormal gait after stroke [7]. These deficits limit the effectiveness of walking training, requiring extensive assistance and constant supervision of therapists to ensure safety during the sessions. Visual-feedback cycling exercise is a safe and economical intervention for lower extremity rehabilitation that provides real-time visual feedback of cycling velocity and load symmetry during exercise. Studies have found that the rhythmic alternating muscle activity during cycling exercise is similar to that of walking, with the timing of muscle group excitation and regulation of motor output intensity modulated by afferent feedback from the lower limbs and extrinsic visual feedback [8], suggesting that visual-feedback cycling exercise and walking are controlled by similar sensorimotor neural networks [9]. Therefore, it is believed that the visual-feedback cycling exercise can improve walking ability by enhancing muscle strength and optimizing motor control [6]. In recent years, the combination of noninvasive brain stimulation (NIBS) and motor training has been regarded as a promising combined protocol to enhance motor function in stroke patients by modulating neural plasticity [10]. Repetitive transcranial magnetic stimulation (rTMS) has emerged as a noninvasive neuromodulation technique with precise spatial targeting, modulating the local neural excitability of selected regions and improving cortical plasticity [11]. High-frequency rTMS applied to the affected motor cortex can increase cortical excitability to improve motor function [12], which has been widely used in poststroke lower limb rehabilitation [13]. Previous research suggested that the combination of rTMS with motor training might mutually maximize their individual effects [10]. The study conducted by Chieffo et al. [14] in stroke patients found that high-frequency rTMS coupled with cycling exercise could improve spasticity and motor function of the lower extremities, supporting the safety and efficacy of the combined approach. In this study, we hypothesized that rTMS coupled with visual-feedback cycling exercise might be an effective method to improve walking ability and stability in stroke patients. Meanwhile, we used single-pulse TMS to evaluate changes in patients' motor cortex excitability to explore the potential mechanisms.

## 2. Materials and Methods

### 2.1. Participants

We recruited stroke patients aged 18–80 from the Department of Rehabilitation Medicine at Sir Run Run Hospital of Nanjing Medical University between January 2024 and May 2024. The inclusion criteria were (1) first-time occurrence of a unilateral supratentorial stroke with unilateral hemiplegia of the lower extremity, at least 1 month prior to the study enrollment, diagnosed by computed tomography (CT) or magnetic resonance imaging (MRI); (2) lower extremity impairment ≥ third level in the Brunnstrom recovery stage (BRS) of the lower extremity; (3) ability to stand independently for at least 10 s and walk independently for more than 2 min without the use of canes or orthoses; (4) ability to follow instructions and complete the study. The exclusion criteria were (1) presence of contraindications to rTMS as suggested in the guidelines [15] (e.g., intracranial implants); (2) lower extremity dysfunction due to causes other than stroke; (3) use of neuropsychotropic drugs, including antidepressants or benzodiazepines; (4) Botox injection within the previous 3 months; (5) severe visual impairment, as well as cardiovascular and pulmonary disease, or advanced diseases affecting other systems. The trial adhered to the principles of the Declaration of Helsinki and was approved by the Ethics Committee of Sir Run Run Hospital, Nanjing Medical University (No. 2023-SR-018). Prior to participation, all patients provided signed informed consent forms. Further details regarding the subjects can be found in Table 1.

### 2.2. Study Design

**Figure 1:**
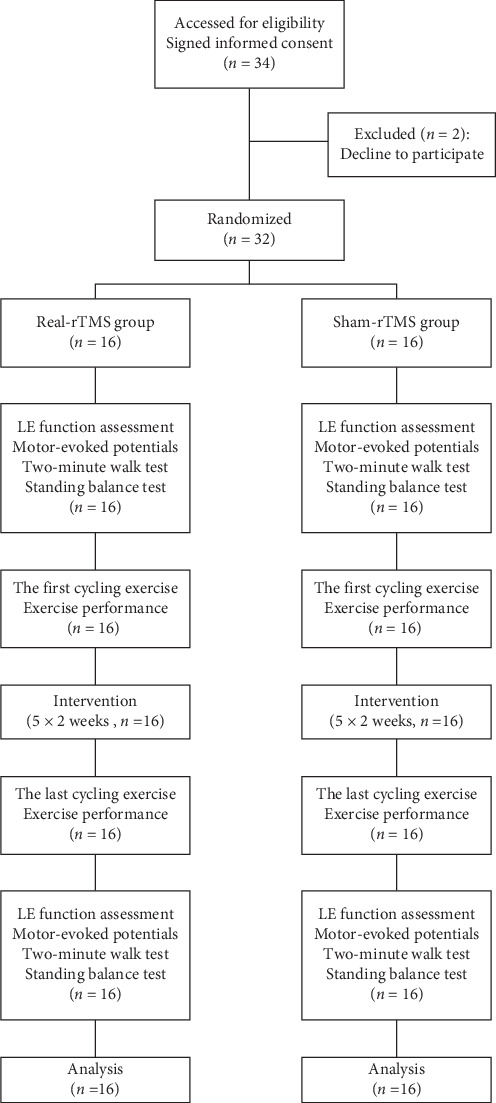
Flow diagram of the study. LE, lower extremity; rTMS, repetitive transcranial magnetic stimulation.

This study was a randomized, double-blind pilot trial that followed the CONSORT checklist, as seen in Supporting Information. The patients were randomized to either the real-rTMS group (RG) or the sham-rTMS group (SG) in a 1:1 ratio, using a random number table. The patients in RG received real rTMS during active visual-feedback cycling exercise, while the patients in SG received sham rTMS during the exercise. The treatment was delivered 5 days per week for 2 weeks. Both groups received conventional training of the same difficulty, involving lower extremity strengthening, walking and balance training. To reduce subjective bias, different individuals performed the roles of the physiotherapist and outcome assessor, while the assessor did not know how the participants were grouped. To assess the cycling exercise performance, the mean velocity and load symmetry values of the initial and final cycling exercises were recorded. The study flow is shown in Figure 1.

### 2.3. Intervention

#### 2.3.1. Visual-Feedback Cycling Exercise

**Figure 2:**
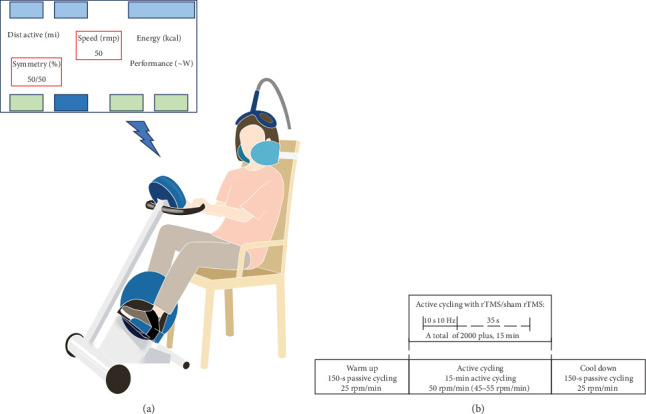
Study design. (A) Experimental setup. (B) Design of the intervention. rTMS, repetitive transcranial magnetic stimulation.

Both groups of patients underwent visual-feedback cycling exercise using the MOTOmed viva2 Movement Trainer (RECK-Technik, Germany). The 20-min visual-feedback cycling exercise consisted of four sessions [16]: (1) Preparation: the patients were seated on a comfortable chair with a high backrest and a head immobilizer to support and stabilize the head, reducing head movement during the exercise. Standardizing the distance from the chair to the crank axis for each participant to ensure a maximum of 110°−120° flexion on the knee joint during cycling. (2) Passive warm-up: a 150-s passive cycling with a constant velocity of 25 rpm. (3) Active cycling: 15-min active cycling with the load set to zero, requiring patients to maintain the cycling cadence at 50 rpm (range: 45–55 rpm) and focus on achieving 50/50 load symmetry of their lower extremities, as displayed on a computer screen in front of the patients. (4) Passive cool-down: a 150-s passive cycling with a constant velocity of 25 rpm. The cycling exercise procedure is presented in Figure 2.

#### 2.3.2. rTMS

rTMS was delivered simultaneously with the 15-min active cycling exercise using the transcranial magnetic stimulator (YRD CCY-I, Wuhan, China) equipped with a double-cone coil. The optimal stimulation site was located on the midsagittal plane, where the largest motor-evoked potentials (MEPs) in the tibialis anterior (TA) muscle of the affected side were elicited on the surface electromyography (sEMG) [17]. If ipsilesional MEPs could not be elicited, the stimulation site was changed to the location where the largest MEPs in unaffected TA muscle were recorded. Stimulation parameters were 10-s trains at 10 Hz and 35-s intertrain intervals, totaling 2000 pulses for 15 min. Stimulation intensity was set at 100% resting motor threshold (RMT) of unaffected TA muscle. RMT was defined as the minimal intensity able to evoke minimal MEPs (≥50 µV in at least 5 out of 10 trials). The center of the coil was positioned vertically over the stimulation site on the midsagittal plane (Figure 2A). Patients in SG received rTMS with a sham coil that did not connect to the stimulator. Besides, recorded sounds similar to those produced by the real coil were provided during exercise [18]. However, since we stimulated the bilateral lower limbs, if the operator observed visual twitches in unaffected lower extremity muscles, the intensity was reduced by 1% increments until the patients were able to complete the cycling exercise [14].

### 2.4. Outcome Measurements

The Fugl-Meyer assessment of lower extremity (FMA-LE) and Berg balance scale (BBS) were used to evaluate the motor and balance function of the hemiplegic lower extremity. The 2-min walk test (2MWT) and standing balance test were employed to evaluate walking and balance ability. The primary outcome measures were the changed scores of FMA-LE and the improvements in walking ability. MEPs were collected to evaluate the effect of rTMS on cortico-spinal excitability. The above outcomes were assessed before the first treatment and after the final treatment. Additionally, cycling exercise performance was recorded after the first and last cycling exercise.

#### 2.4.1. Cycling Exercise Performance

Cycling exercise performance mainly includes the mean velocity and load symmetry of bilateral limbs retrieved from the bike chip after the session, which can directly evaluate motor control when visual feedback is provided [19]. Load symmetry refers to the percentage of exertion in the affected limb to the sound limb, and perfect symmetry tends to be 50/50 (100%). The mean velocity involves the average pedaling velocity along with the standard deviation (SD). Furthermore, we calculated the cycling asymmetry and velocity variability, as written in Section 2.5.

#### 2.4.2. Lower Extremity Function Assessment

The FMA-LE is a widely used tool to evaluate lower extremity impairment and predict recovery of motor function in stroke patients [20]. The FMA-LE comprises 17 items with a total score of 34 points, evaluating the performance of motor movement, reflex activities, and coordination on the lower limbs [21].

The BBS is an extensively used observational scale to assess balance and risk for falls in stroke survivors [22]. The BBS consists of 14 items, with a maximum score of 56 points. A higher score indicates better balance control and a lower risk of falling [23].

#### 2.4.3. Walking and Balance Ability Assessment

Walking and balance data were obtained using a wireless ambulatory Parkinson's disease monitoring (APDM) movement monitoring inertial sensor system (APDM Inc., Portland, OR, USA) during the 2MWT and standing balance test. After recalibration, six synchronized opal inertial sensors were fitted on the sternum, waist, dorsal surface of bilateral wrists, and top of each foot on each patient (sensor placement, Supporting Information). The signals were processed and calculated via the corresponding Mobility Lab software package [24].

The 2MWT is an easy and sensitive test with a high completion rate in stroke patients, effectively assessing exercise tolerance and gait patterns [25]. The objective of the test was to walk as far as possible for 2 min at a comfortable and safe speed. We chose certain temporal and spatial gait parameters for further analysis, as written in Section 2.5.

The standing balance test is a safe and common test to evaluate postural control, which is important to walking ability [26, 27]. Patients were required to fit their feet around the foot template to normalize foot placement on the solid surface, then stand comfortably with their hands at their sides and try their best to stay still [28]. Each patient underwent the test twice, and each test lasted 10 s, followed by a 30-s rest. Temporal and spatial parameters of posture sway were measured, and mean values were calculated for analysis.

#### 2.4.4. MEPs Examination

MEPs are the action potentials elicited by single-pulse TMS of the motor cortex, which can assess the integrity of motor pathways [29]. In this study, we used MEPs as indicators of motor cortical excitability in stroke patients, consistent with previous studies [30, 31]. Our MEPs examination procedure followed established practice guidelines [32]. The center of the double-cone coil was placed over the primary motor cortex of the lower limbs to elicit the MEPs, while sEMG recorded electrical signals from the TA muscle. The initial intensity was set at 10% of the maximum stimulator output (MSO) and gradually increased in 5% increments until the minimal MEPs were recorded. We collected the average latency and amplitude of the bilateral MEPs [33]. The processing pipeline for MEPs data can be found in Supporting Information. If MEPs could not be elicited at 100% MSO, the result was recorded as “not applicable (NA).”

### 2.5. Data Processing

We calculated cycling asymmetry to evaluate the load asymmetry of bilateral lower extremities during cycling [34]:

  $$\begin{array}{c} \text{Cycling asymmetry }=\frac{\left|V\text{unaffected}-V\text{affected}\right|}{V\text{unaffected}}. \end{array}$$

To eliminate the confounding impact of body height, we normalized cadence, walking speed, and stride length [24]:

  $$\begin{array}{c} \text{Normalized cadence}=\text{cadence}\times \text{sqrt}\left(\frac{\text{body height}}{\text{mean body height}}\right), \end{array}$$

  $$\begin{array}{c} \text{Normalized speed}=\text{speed}/\text{sqrt}\left(\frac{\text{body height}}{\text{mean body height}}\right), \end{array}$$

  $$\begin{array}{c} \text{Normalized stride length}=\text{stride length}/\text{sqrt}\left(\frac{\text{body height}}{\text{mean body height}}\right). \end{array}$$

We calculated the absolute symmetry index (ASI) of the stance phase, swing phase, and step length of both sides to evaluate the degree of temporal and spatial asymmetry in walking [35]:

  $$\begin{array}{c} \text{ASI}=\frac{\left|V\text{unaffected}-V\text{affected}\right|}{0.5\left(V\text{unaffected}+V\text{affected}\right)}. \end{array}$$

The coefficients of variation (CV) of cadence, speed, stride length, and gait cycle duration were calculated to evaluate walking stability [36, 37]. Additionally, we calculated the CV of cycling velocity to assess motor control during visual-feedback cycling:

  $$\begin{array}{c} \text{CV}=\frac{\text{standard deviation}}{\text{mean}}\times 100\%. \end{array}$$

### 2.6. Statistical Analysis

The necessary sample size was calculated using the PASS15.0 software based on the previous study, seen in Supporting Information. The statistical analysis was conducted using the statistical software package SPSS 22.0 (SPSS, Inc., Chicago, IL, USA). The complete-case analysis was used to handle the missing data. Thus, participants who dropped out would be excluded from the statistical analysis. The Shapiro–Wilks test was conducted to assess the normality of the data. Baseline demographic and clinical characteristics were analyzed using the Fisher's exact test for categorical variables, while the two-sample *t*-test and Mann–Whitney test were applied for continuous variables. For the FMA-LE and BBS scores, as well as the parameters of 2WMT and standing balance test, the paired *t*-test was used to analyze within-group differences pre- and posttreatment. The between-group comparison was conducted using the independent *t*-test, with Cohen's *d* used to determine effect sizes (effect sizes of <0.5, 0.5–0.8, and >0.8 were considered small, moderate, and large, respectively [38]). Given the impairment of motor pathway integrity after stroke, the statistical analysis for MEPs was determined based on the number of MEPs collected. Pearson correlation analysis was utilized to evaluate the correlation between the changes in cycling exercise performance, the recovery of lower extremity function, and the improvements in walking and balance ability. Furthermore, a linear regression analysis was performed to analyze the correlation between improvements in cycling asymmetry and changes in other measures. The statistical significance levels for all tests were set at *p*  < 0.05.

## 3. Results

Thirty-four patients with stroke were enrolled, while two participants dropped out of the study due to unwillingness. Therefore, 32 patients were randomly allocated to the RG and SG. The flowchart can be found in Figure 1. All participants were able to complete the study interventions and assessments, and no adverse events were reported throughout the study. No significant between-group differences were found in pretreatment regarding all primary and secondary outcomes (*p* > 0.05).

### 3.1. Demographics and Baseline Clinical Characteristics

The results of the demographics and baseline clinical characteristics can be found in Table 1. There were no statistically significant differences between the two groups in any of the variables, including age, gender, type of stroke, affected side, duration of stroke, height, and BSS of individuals.

### 3.2. Outcomes for Cycling Exercise Performance

Compared to the baseline, the patients in both groups showed significant improvements in cycling asymmetry. However, RG showed statistically greater improvements than SG (*p* = 0.001, 95% confidence interval [CI] [−0.120, −0.039], Table 2), demonstrating greater facilitation of motor control. Besides, after the treatment, the CV of cycling velocity decreased from 11% to 7.18% in RG, while it increased from 9.83% to 12.25% in SG (Table 2).

### 3.3. Outcomes for Lower Extremity Function

**Figure 3:**
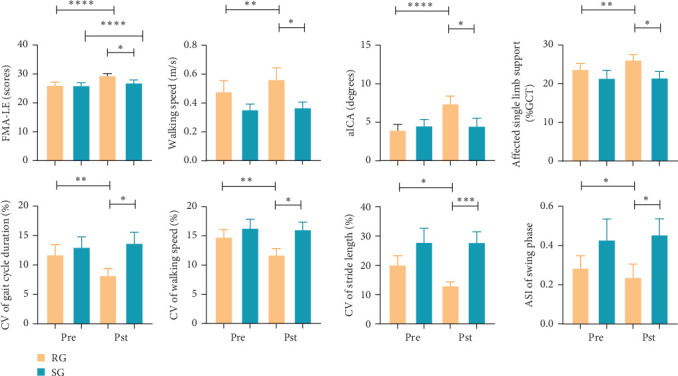
Primary outcomes. aICA, affected initial contact angle; ASI, absolute symmetry index; CV, coefficients of variation; FMA-LE, Fugl-Meyer assessment of lower extremity; GCT, gait cycle time; Pre, pretreatment; Pst, posttreatment; RG, real-rTMS group; SG, sham-rTMS group. ⁣^*∗∗∗∗*^: *p*  < 0.0001; ⁣^*∗∗∗*^: *p*  < 0.001; ⁣^*∗∗*^: *p*  < 0.01; ⁣^*∗*^: *p*  < 0.05.

After the treatment, the lower extremity function significantly improved in RG compared to SG (FMA-LE: *p* = 0.045, 95% CI [0.059, 4.941]; BBS: *p* = 0.019, 95% CI [0.814, 8.561]; Figure 3, Table 3).

### 3.4. Outcomes for Walking and Balance Ability

The gait characteristics are listed in Table 4. The walking ability and stability showed marked improvements in RG compared to SG. The results found that walking speed (*p* =  0.034, 95% CI [0.016, 0.378]), stride length (*p* = 0.042, 95% CI [0.007, 0.362]), affected single limb support phase (*p* = 0.042, 95% CI [0.188, 9.074]), and affected initial contact angle (ICA) (*p* = 0.048, 95% CI [0.021, 5.820]) significantly increased after treatment in RG than those in SG. There were statistically significant reductions in gait cycle duration (*p* = 0.040, 95% CI [−0.599, −0.015]) and affected step duration (*p* = 0.009, 95% CI [−0.454, −0.073]) in RG. Besides, after treatment, the CV of gait cycle duration (*p* = 0.017, 95% CI [−9.932, −1.031]), walking speed (*p* = 0.014, 95% CI [−7.661, −0.945]), stride length (*p* = 0.001, 95% CI [−22.669, −6.960]), as well as the ASI of swing phase (*p* = 0.048, 95% CI [−0.431, −0.002]) in RG significantly reduced compared to SG (Figure 3).

For the standing balance test, the parameters of postural sway in RG significantly decreased compared to baseline, while such significance was not observed in SG (Table 5). Posttreatment, significant intergroup differences were observed in 95% ellipse sway area (*p* = 0.010, 95% CI [−0.098, −0.015]), root mean square (RMS) sway (*p* = 0.003, 95% CI [−0.078, −0.018]), sway path length (*p* = 0.008, 95% CI [−7.790, −1.301]), and sway range (*p* = 0.008, 95% CI [−0.343, −0.056]).

### 3.5. Outcomes for MEPs

In our study, only eight patients could elicit ipsilesional MEPs at baseline (RG, *n* = 5; SG, *n* = 3). After the treatment, the number of patients who were able to elicit ipsilesional MEPs increased to 16 (RG,*n* = 12; SG, *n* = 4). Therefore, we utilized Fisher's exact test for between-group comparison and the McNemar test for within-group comparison to explore the effects on the elicitation rate of ipsilesional MEPs [31]. The RG showed a significantly higher elicitation rate of ipsilesional MEPs than that in both the baseline (*p* = 0.016) and the SG (*p* = 0.012, Table 6). No significant changes in contralesional MEPs were detected in the two groups.

### 3.6. Outcomes for Correlation

**Figure 4:**
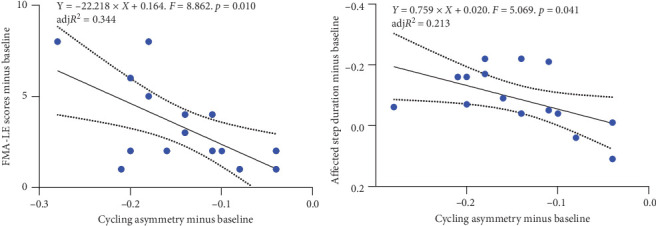
Correlations between cycling performance and lower extremity function/walking ability. FMA-LE: Fugl-Meyer assessment of lower extremity.

The linear regression model analysis revealed that the reductions in cycling asymmetry were significantly related to the improvements in FMA-LE (adj*R*^2^ = 0.344, *F* = 8.862, *p* = 0.010) and the decrease in affected step duration (adj*R*^2^ = 0.213, *F* = 5.069, *p* = 0.041) in RG, as shown in Figure 4. The correlation matrix of significant variables in RG is represented in Table 7. The results showed that the decrease in sway path length was positively correlated with the reduction in gait cycle duration (*r* = 0.532, *p* = 0.034), while it was negatively correlated with the increase in affected single limb support phase (*r* = −0.566, *p* = 0.022). Additionally, the decline in CV of walking speed was positively related to the diminution in ellipse sway area (*r* = 0.516, *p* = 0.041) and RMS sway (*r* = 0.586, *p* = 0.017).

## 4. Discussion

To the best of our knowledge, our study is the first to explore the effects of NIBS coupled with visual-feedback cycling exercise on the walking ability and stability of stroke patients. Previous studies found that cycling with visual feedback could improve neuromuscular control and motor performance in the lower extremities [16]. In this study, we observed a significant decrease in cycling asymmetry in RG compared to SG, indicating that rTMS coupled with visual-feedback cycling exercise could further enhance motor control and cycling exercise performance. The results showed that both groups improved in lower extremity motor capacity (FMA-LE) and balance ability (BBS), while patients in RG demonstrated markedly greater improvements. Besides, we found a significant correlation between the decrease in cycling asymmetry and the increase in FMA-LE scores (*r* = −0.623), which was consistent with previous literature [39]. It has been reported that the NIBS simultaneously applied during motor training might preferentially interact with the neural networks selectively recruited by the ongoing exercise [10]. Based on these findings, it is plausible to speculate that the additional rTMS applied during the visual-feedback cycling exercise can enhance exercise performance by activating the neural networks involved in cycling, increasing the functional benefits for lower extremities.

In this study, we utilized the 2MWT to assess the effects of rTMS coupled with visual-feedback cycling exercise on abnormal gait in stroke patients. Individuals with stroke demonstrate a decline in walking ability due to impaired motor function of the hemiplegic lower limb, including reduced walking speed, prolonged gait cycle duration, shortened stride length, and decreased single limb support phase of the affected side [40]. Our study showed significant increases in walking speed and stride length, with marked decreases in gait cycle duration by reducing the step duration of the affected limb in RG. These results indicated that the combination of rTMS and visual-feedback cycling exercise could significantly improve walking efficiency in stroke patients. In addition to these fundamental gait parameters, we also focused on the characteristics that reflected impairments in foot trajectory control during the swing phase. Specifically, the ICA refers to the angle between the foot and the ground when the foot initially hits the ground, reflecting the rapid dorsiflexion movement of the ankle during the swing phase [24, 41]. Notably, stroke patients with gait dysfunction experience limited dorsiflexion movement of the hemiplegic ankle when walking, predisposing them to greater risks of falling [42]. Wada et al. [43] reported a significant correlation between the increased ankle dorsiflexion angle during the swing phase and the reduced risk of falling in stroke patients. In the RG, we found a significant increase in ICA of the affected ankle, indicating that the combination intervention could effectively enhance affected ankle dorsiflexion movement during walking. Additionally, patients with hemiplegia tend to develop a compensatory gait pattern for strength deficits of the paretic limb [44]. This requires the unaffected limb to provide more power and balance support when walking, resulting in worse gait symmetry, poor dynamic balance, and increased gait variability [45]. The marked increase in the single limb support phase of the affected side in RG suggested that the combination of rTMS and visual-feedback cycling exercise could enhance the weight bearing on the paretic limb and improve coordination between lower limbs when walking [46]. The RG showed a significant decrease in ASI of the swing phase, along with notable reductions in the CV of gait cycle duration, walking speed, and stride length. These results indicated that rTMS coupled with visual-feedback cycling exercise effectively reduced gait asymmetry and gait variability, thereby enhancing walking stability. Studies suggest that cycling exercise alternatively activates agonist and antagonist muscles, requiring reciprocal flexion and extension movements of the hip, knee, and ankle, which activate similar kinematic patterns and neural networks to those involved in walking and modulate motor functional reorganization [17, 19]. The positive correlations between the decrease in cycling exercise asymmetry and the reduction in step duration of the hemiplegic limb seen in RG support this idea. Besides, previous systematic reviews and meta-analyses indicated that rTMS effectively improved gait function by boosting activity-dependent plasticity in stroke patients [10, 47]. Based on these findings, we speculated that rTMS could enhance functional recovery by activating the related neural pathway and improving neural plasticity.

Balance is one of the fundamental components of walking ability [27]. More than half of stroke patients experience balance dysfunction, restricting their walking ability [48]. Recently, studies in stroke patients suggest that the combination of rTMS and motor training can effectively improve postural control [49, 50]. Our study found that the standing postural sway of patients significantly diminished in RG. Notably, the parameters of standing postural sway in the coronal plane were significantly changed, while no marked difference was observed in the sagittal plane in this study. It is reported that individuals with hemiplegic lower limbs need more time to adjust their posture to maintain balance in the coronal plane and to prepare for the subsequent step when walking [51]. Therefore, disequilibrium in the coronal plane has been regarded as an important predictor of unstable gait and falls [52]. For stroke patients, it not only decreases walking efficiency but also limits their ability to adapt to different environments and respond to unexpected balance perturbations [46]. Besides, a significant correlation between the improvements in standing balance and the reduction in gait cycle duration was observed in our study. These findings provide evidence that rTMS coupled with visual-feedback cycling exercise can effectively improve standing balance, thereby enhancing walking efficiency and stability.

Additionally, we considered MEPs as the indicators to evaluate the changes in motor cortex excitability. After treatment, the elicitation rate of ipsilesional MEPs significantly increased from 31.25% to 75% in RG, while no marked change was observed in SG. A randomized controlled trial [53] demonstrated that the increased elicitation rate of ipsilesional MEPs could predict better recovery of lower limb motor function. Besides, in the RG, the average latency of ipsilesional MEPs decreased from 36.23 to 34.69 ms, while the average amplitude increased from 59.28 to 93.80 µV. These results indicated a trend toward enhanced cortical excitability in the ipsilesional M1, consistent with the findings of Sivaramakrishnan and Madhavan [54]. Stimulation-primed motor training can strengthen synaptic connections within M1, facilitating the processing and storage of new information for motor consolidation [55]. Combining these findings with the results of our study, it is reasonable to speculate that the high-frequency rTMS can improve task-specific cortical plasticity by increasing the motor cortex excitability during cycling exercise [10], thereby improving functional reorganization [12].

Several limitations of this study need to be addressed in future research. First, this pilot study aimed to examine the feasibility and effectiveness of the combination intervention in stroke rehabilitation, which could inform and direct future studies. However, by the very nature of pilot studies, there were critical limitations to their role and interpretation. Especially, the small sample size in our study resulted in insufficient statistical power and a limited number of positive events (elicited ipsilesional MEPs), weakening the validation of these findings. Second, as most participants were patients with chronic stroke, the applicability of the results to subacute stroke patients needed further consideration. Third, we only recorded the electrophysiological parameters (MEPs) but did not obtain the functional brain imaging data. Fourth, no follow-up assessments were conducted in our study. Therefore, future studies need to increase the sample size to confirm current findings and achieve more reliable results, utilize functional MRI and functional near-infrared spectroscopy to further explore the potential mechanisms of the combination treatment, and conduct follow-up assessments to investigate the sustained effects.

## 5. Conclusion

In summary, the results of this study provided evidence that the combination of rTMS and visual-feedback cycling exercise could effectively improve walking ability and walking stability in patients with stroke. These improvements could be attributed to the increased motor cortex excitability induced by high-frequency rTMS, which improved neural plasticity and facilitated motor functional reorganization. It was recommended that rTMS coupled with visual-feedback cycling exercise could be considered a clinical protocol in stroke patients with gait dysfunction.

## Figures and Tables

**Table 1:** Demographics and baseline clinical characteristics.

Characteristics	Real-rTMS group	Sham-rTMS group	*p*-Value
Age (year)	52.38 ± 11.94	47.94 ± 18.39	0.425
Gender (*n*, male/female)	4/12	6/10	0.704^F^
Type of stroke (*n*, infarction/hemorrhage)	9/7	6/10	0.479^F^
Affected side (*n*, left/right)	8/8	7/9	*p*＞0.999^F^
Duration (month)	8.50 ± 7.74	8.47 ± 7.88	0.991
Height (m)	1.68 ± 0.08	1.69 ± 0.06	0.771
Brunnstrom stages (III/IV/V)	4/8/4	3/9/4	0.803^U^

Abbreviations: F, Fisher's exact test; U, Mann–Whitney *U* test.

**Table 2:** Outcomes for cycling exercise performance.

	Pretreatment			Posttreatment		
	RG (mean ± SD)	SG (mean ± SD)	*p*-Value	RG (mean ± SD)	SG (mean ± SD)	*p*-Value (effect size)
Cycling asymmetry	0.19 ± 0.09	0.18 ± 0.09	0.635	0.05 ± 0.03⁣^*∗*^	0.13 ± 0.07⁣^*∗*^	0.001 (1.431)
Mean velocity (rpm)	48.13 ± 3.46	47.69 ± 3.88	0.739	49.56 ± 2.07	49.00 ± 5.14	0.563 (0.142)
CV of mean velocity (%)	7.19	8.13	/	4.17	10.49	/

Abbreviations: CV, coefficients of variation; RG, real-rTMS group; SD, standard deviation; SG, sham-rTMS group.

⁣^*∗*^ Significant difference between pre- and posttreatment, *p*  < 0.05.

**Table 3:** Outcomes for lower extremity function.

	Pretreatment			Posttreatment		
	RG (mean ± SD)	SG (mean ± SD)	*p*-Value	RG (mean ± SD)	SG (mean ± SD)	*p*-Value (effect size)
FMA-LE	26.13 ± 4.16	26.00 ± 3.90	0.931	29.44 ± 2.66⁣^*∗*^	26.94 ± 3.97⁣^*∗*^	0.045 (0.721)
BBS	45.06 ± 6.97	43.00 ± 6.06	0.379	49.31 ± 5.19⁣^*∗*^	44.63 ± 5.54⁣^*∗*^	0.019 (0.850)

Abbreviations: BBS, Berg balance scale; FMA-LE, Fugl-Meyer assessment of lower extremity; RG, real-rTMS group; SD, standard deviation; SG, sham-rTMS group.

⁣^*∗*^ Significant difference between pre- and posttreatment, *p*  < 0.05.

**Table 4:** Outcomes for 2-min walk test.

	Pretreatment			Posttreatment		
	RG (mean ± SD)	SG (mean ± SD)	*p*-Value	RG (mean ± SD)	SG (mean ± SD)	*p*-Value (effect size)
Cadence (steps/min)^n^	81.73 ± 17.75	75.44 ± 21.04	0.368	86.85 ± 17.24⁣^*∗*^	74.80 ± 20.08	0.079 (0.628)
Speed (m/s)^n^	0.48 ± 0.31	0.35 ± 0.16	0.156	0.57 ± 0.32⁣^*∗*^	0.37 ± 0.15	0.034 (0.780)
Stride length (m)^n^	0.67 ± 0.30	0.58 ± 0.19	0.298	0.79 ± 0.28⁣^*∗*^	0.61 ± 0.21	0.042 (0.709)
Gait cycle duration (s)	1.58 ± 0.41	1.78 ± 0.56	0.265	1.46 ± 0.31⁣^*∗*^	1.76 ± 0.48	0.040 (0.723)
Affected step duration (s)	0.85 ± 0.23	1.05 ± 0.52	0.173	0.77 ± 0.19⁣^*∗*^	1.03 ± 0.32	0.009 (0.963)
Affected foot clearance (cm)	2.36 ± 1.21	3.02 ± 2.00	0.266	2.67 ± 1.52	3.24 ± 2.03	0.380 (0.310)
Affected single limb support (%GCT)	23.74 ± 5.83	21.44 ± 8.19	0.367	26.14 ± 5.57⁣^*∗*^	21.51 ± 6.69	0.042 (0.733)
Affected swing phase (%GCT)	31.95 ± 7.03	34.63 ± 10.94	0.417	32.05 ± 6.18	35.04 ± 11.02	0.353 (0.327)
Double support phase (%GCT)	45.82 ± 10.11	44.32 ± 14.27	0.735	42.14 ± 9.59	45.32 ± 13.45	0.447 (0.265)
Affected initial contact angle (degree)	4.00 ± 2.92	4.55 ± 3.19	0.618	7.43 ± 3.94⁣^*∗*^	4.50 ± 4.09	0.048 (0.712)
Affected toe-off angle (degree)	25.90 ± 15.33	20.60 ± 8.11	0.234	30.92 ± 16.58⁣^*∗*^	22.84 ± 8.76	0.098 (0.594)
Coefficient of variation (%)						
Cadence	9.30 ± 4.61	12.22 ± 5.37	0.109	7.90 ± 4.15	11.27 ± 4.95	0.046 (0.719)
Gait cycle duration	11.76 ± 6.72	13.04 ± 6.92	0.601	8.23 ± 4.60⁣^*∗*^	13.72 ± 7.41	0.017 (0.868)
Speed	14.82 ± 5.09	16.33 ± 6.17	0.456	11.79 ± 4.18⁣^*∗*^	16.09 ± 5.08	0.014 (0.901)
Stride length	20.23 ± 12.11	27.91 ± 18.85	0.182	13.07 ± 5.12⁣^*∗*^	27.89 ± 14.11	0.001 (1.361)
Absolute symmetry index						
Swing phase	0.29 ± 0.25	0.44 ± 0.42	0.253	0.24 ± 0.27⁣^*∗*^	0.46 ± 0.32	0.048 (0.724)
Stance phase	0.12 ± 0.10	0.19 ± 0.19	0.243	0.11 ± 0.11	0.20 ± 0.17	0.074 (0.613)
Step length	0.06 ± 0.05	0.11 ± 0.09	0.055	0.05 ± 0.08	0.09 ± 0.08	0.212 (0.487)

Abbreviations: GCT, gait cycle time; n, normalization; RG, real-rTMS group; SD, standard deviation; SG, sham-rTMS group.

⁣^*∗*^ Significant difference between pre- and posttreatment, *p*  < 0.05.

**Table 5:** Outcomes for standing balance test.

	Pretreatment			Posttreatment		
	RG (mean ± SD)	SG (mean ± SD)	*p*-Value	RG (mean ± SD)	SG (mean ± SD)	*p*-Value (effect size)
95% Ellipse sway area (m^2^/s^4^)	0.072 ± 0.063	0.079 ± 0.068	0.738	0.034 ± 0.036⁣^*∗*^	0.091 ± 0.072	0.010 (0.976)
RMS sway (m/s^2^)	0.095 ± 0.055	0.092 ± 0.034	0.871	0.060 ± 0.029⁣^*∗*^	0.108 ± 0.051	0.003 (1.128)
RMS sway (coronal) (m/s^2^)	0.066 ± 0.035	0.070 ± 0.032	0.723	0.042 ± 0.020⁣^*∗*^	0.080 ± 0.037	0.001 (1.188)
RMS sway (sagittal) (m/s^2^)	0.057 ± 0.057	0.060 ± 0.029	0.876	0.036 ± 0.031	0.064 ± 0.043	0.044 (0.728)
Path length (m/s^2^)	9.666 ± 6.759	10.301 ± 5.839	0.778	7.357 ± 4.214⁣^*∗*^	11.903 ± 4.757	0.008 (0.986)
Path length (coronal) (m/s^2^)	5.851 ± 4.387	6.160 ± 2.777	0.814	4.261 ± 2.909⁣^*∗*^	6.879 ± 2.977	0.017 (0.867)
Path length (sagittal) (m/s^2^)	6.461 ± 4.386	7.581 ± 3.855	0.449	5.034 ± 2.629	8.279 ± 3.259	0.004 (1.068)
Range (m/s^2^)	0.455 ± 0.229	0.461 ± 0.221	0.944	0.311 ± 0.201⁣^*∗*^	0.511 ± 0.197	0.008 (0.980)
Range (coronal) (m/s^2^)	0.255 ± 0.161	0.253 ± 0.118	0.969	0.137 ± 0.103⁣^*∗*^	0.273 ± 0.131	0.003 (1.125)
Range (sagittal) (m/s^2^)	0.377 ± 0.189	0.375 ± 0.206	0.986	0.272 ± 0.185	0.423 ± 0.173	0.023 (0.822)

Abbreviations: RG, real-rTMS group; RMS, root mean square; SD, standard deviation; SG, sham-rTMS group.

⁣^*∗*^ Significant difference between pre- and posttreatment, *p*  < 0.05.

**Table 6:** Outcomes for motor-evoked potentials.

	Pretreatment			Posttreatment		
	RG (mean ± SD)	SG (mean ± SD)	*p*-Value	RG (mean ± SD)	SG (mean ± SD)	*p*-Value (effect size)
Contralesional						
Latency (ms)	31.11 ± 1.97	29.24 ± 4.26	0.213	29.68 ± 1.89	30.18 ± 3.52	0.697 (0.173)
Amplitude (μV)	165.11 ± 102.68	182.65 ± 111.02	0.744	250.18 ± 260.00	142.47 ± 55.28	0.337 (0.559)
Ipsilesional						
Elicited (*n*)	5	3	0.657^F^	12^M^⁣^*∗*^	4	0.012^F^
Not elicited (*n*)	11	13		4	12	

Abbreviations: F, Fisher's exact test; M, McNemar test; RG, real-rTMS group; SD, standard deviation; SG, sham-rTMS group.

⁣^*∗*^ Significant difference between pre- and posttreatment, *p*  < 0.05.

**Table 7:** Correlation matrix.

	CAS	FMA-LE	BBS	GCD	aSD	aSLS	CV (speed)	RMS sway	95% Ellipse sway area	Path length	Path length (coronal)
CAS	1.000	−0.623⁣^*∗∗*^	—	—	0.516⁣^*∗*^	—	—	—	—	—	—
FMA-LE	−0.623⁣^*∗∗*^	1.000	0.578⁣^*∗*^	—	—	—	—	—	−0.539⁣^*∗*^	—	—
BBS	—	0.578⁣^*∗*^	1.000	—	—	—	—	−0.526⁣^*∗*^	−0.816⁣^*∗∗*^	—	—
GCD	—	—	—	1.000	0.709⁣^*∗∗*^	−0.808⁣^*∗∗*^	—	—	—	0.532⁣^*∗*^	0.513⁣^*∗*^
aSD	0.516⁣^*∗*^	—	—	0.709⁣^*∗∗*^	1.000	−0.631⁣^*∗∗*^	—	—	—	—	—
aSLS	—	—	—	−0.808⁣^*∗∗*^	−0.631⁣^*∗∗*^	1.000	—	—	—	−0.566⁣^*∗*^	−0.568⁣^*∗*^
CV (speed)	—	—	—	—	—	—	1.000	0.586⁣^*∗*^	0.516⁣^*∗*^	—	—
RMS sway	—	—	−0.526⁣^*∗*^	—	—	—	0.586⁣^*∗*^	1.000	0.732⁣^*∗∗*^	—	0.687⁣^*∗∗*^
95% Ellipse sway area	—	−0.539⁣^*∗*^	−0.816⁣^*∗∗*^	—	—	—	0.516⁣^*∗*^	0.732⁣^*∗∗*^	1.000	0.498⁣^*∗*^	0.512⁣^*∗*^
Path length	—	—	—	0.532⁣^*∗*^	—	−0.566⁣^*∗*^	—	—	0.498⁣^*∗*^	1.000	—
Path length (coronal)	—	—	—	0.513⁣^*∗*^	—	−0.568⁣^*∗*^	—	0.687⁣^*∗∗*^	0.512⁣^*∗*^	—	1.000

*Note:* Only significant Pearson's correlation coefficients (*p*  < 0.05) are presented.

Abbreviations: aSD, affected step duration; aSLS, affected single limb support; BBS, Berg balance scale; CAS, cycling asymmetry; CV, coefficients of variation; FMA-LE, Fugl-Meyer assessment of lower extremity; GCD, gait cycle duration; RMS, root mean square.

⁣^*∗∗*^*p*  < 0.001; ⁣^*∗*^*p*  < 0.05.

## Data Availability

The data that support the findings of this study are available from the corresponding author upon reasonable request.

## Supporting Information

Supporting Information Refer to Supporting Information for supporting information mentioned in the manuscript, including the CONSORT checklist, sample size calculation, sensor placement, and the processing pipeline for MEPs data.
