# Supplementary material for: Repetitive Transcranial Magnetic Stimulation Coupled With Visual-Feedback Cycling Exercise Improves Walking Ability and Walking Stability After Stroke: A Randomized Pilot Study
Source: Neural Plast. 2024 Nov 26;2024:8737366. doi: 10.1155/np/8737366 (PMC11614519; doi:10.1155/np/8737366)
Supplement: Supporting Information — Refer to Supporting Information for supporting information mentioned in the manuscript, including the CONSORT checklist, sample size calculation, sensor placement, and the processing pipeline for MEPs data. [file 8737366.f1.docx]

# Supplementary Material

CONSORT 2010 checklist of information to include when reporting a randomized trial*

| Section/Topic | Item No | Checklist item | Reported on page No |
| --- | --- | --- | --- |
| Title and abstract | | | |
|  | 1a | Identification as a randomized trial in the title | P1 |
|  | 1b | Structured summary of trial design, methods, results, and conclusions (for specific guidance see CONSORT for abstracts) | P1 |
| Introduction | | | |
| Background and objectives | 2a | Scientific background and explanation of rationale | P2 |
|  | 2b | Specific objectives or hypotheses | P2 |
| Methods | | | |
| Trial design | 3a | Description of trial design (such as parallel, factorial) including allocation ratio | P3 |
|  | 3b | Important changes to methods after trial commencement (such as eligibility criteria), with reasons | Not need |
| Participants | 4a | Eligibility criteria for participants | P2-3 |
|  | 4b | Settings and locations where the data were collected | P3 |
| Interventions | 5 | The interventions for each group with sufficient details to allow replication, including how and when they were actually administered | P3-4 |
| Outcomes | 6a | Completely defined pre-specified primary and secondary outcome measures, including how and when they were assessed | P4-5 |
|  | 6b | Any changes to trial outcomes after the trial commenced, with reasons | Not need |
| Sample size | 7a | How sample size was determined | Supplementary |
|  | 7b | When applicable, explanation of any interim analyses and stopping guidelines | Not need |
| Randomisation: |  |  |  |
| Sequence  generation | 8a | Method used to generate the random allocation sequence | P3 |
|  | 8b | Type of randomisation; details of any restriction (such as blocking and block size) | P3 |
| Allocation  concealment mechanism | 9 | Mechanism used to implement the random allocation sequence (such as sequentially numbered containers), describing any steps taken to conceal the sequence until interventions were assigned | P3 |
| Implementation | 10 | Who generated the random allocation sequence, who enrolled participants, and who assigned participants to interventions | P3 |
| Blinding | 11a | If done, who was blinded after assignment to interventions (for example, participants, care providers, those assessing outcomes) and how | P3 |
|  | 11b | If relevant, description of the similarity of interventions | P3 |
| Statistical methods | 12a | Statistical methods used to compare groups for primary and secondary outcomes | P6 |
|  | 12b | Methods for additional analyses, such as subgroup analyses and adjusted analyses | Not need |
| Results | | | |
| Participant flow (a diagram is strongly recommended) | 13a | For each group, the numbers of participants who were randomly assigned, received intended treatment, and were analysed for the primary outcome | P6, Figure 1 |
|  | 13b | For each group, losses and exclusions after randomisation, together with reasons | P6, Figure 1 |
| Recruitment | 14a | Dates defining the periods of recruitment and follow-up | P2 |
|  | 14b | Why the trial ended or was stopped | Not need |
| Baseline data | 15 | A table showing baseline demographic and clinical characteristics for each group | Table 1 |
| Numbers analysed | 16 | For each group, number of participants (denominator) included in each analysis and whether the analysis was by original assigned groups | P6, Figure 1 |
| Outcomes and estimation | 17a | For each primary and secondary outcome, results for each group, and the estimated effect size and its precision (such as 95% confidence interval) | P6-7, Table 2-7 |
|  | 17b | For binary outcomes, presentation of both absolute and relative effect sizes is recommended | None |
| Ancillary analyses | 18 | Results of any other analyses performed, including subgroup analyses and adjusted analyses, distinguishing pre-specified from exploratory | Not need |
| Harms | 19 | All-important harms or unintended effects in each group (for specific guidance see CONSORT for harms) | None |
| Discussion | | | |
| Limitations | 20 | Trial limitations, addressing sources of potential bias, imprecision, and, if relevant, multiplicity of analyses | P9 |
| Generalisability | 21 | Generalisability (external validity, applicability) of the trial findings | P7-9 |
| Interpretation | 22 | Interpretation consistent with results, balancing benefits and harms, and considering other relevant evidence | P7-9 |
| Other information | | |  |
| Registration | 23 | Registration number and name of trial registry | P3 |
| Protocol | 24 | Where the full trial protocol can be accessed, if available | None |
| Funding | 25 | Sources of funding and other support (such as supply of drugs), role of funders | P10 |

Citation: Schulz KF, Altman DG, Moher D, for the CONSORT Group. CONSORT 2010 Statement: updated guidelines for reporting parallel group randomised trials. BMC Medicine. 2010;8:18.

© 2010 Schulz et al. This is an Open Access article distributed under the terms of the Creative Commons Attribution License (<http://creativecommons.org/licenses/by/2.0>), which permits unrestricted use, distribution, and reproduction in any medium, provided the original work is properly cited.

*We strongly recommend reading this statement in conjunction with the CONSORT 2010 Explanation and Elaboration for important clarifications on all the items. If relevant, we also recommend reading CONSORT extensions for cluster randomised trials, non-inferiority and equivalence trials, non-pharmacological treatments, herbal interventions, and pragmatic trials. Additional extensions are forthcoming: for those and for up-to-date references relevant to this checklist, see [www.consort-statement.org](http://www.consort-statement.org).

**Sample size calculation:**

To determine the sample size for our study, we calculated it using the analysis of two-sample t test in PASS15.0 software. The effect size was calculated with reference to the research results of Chieffo et al(Chieffo et al., 2021), using FMA-LE as the measurement index. Assuming a target power of 0.80, the type I error level at 0.05, and considering a 20% dropout rate, it was determined that each group needed at least 15 subjects, resulting in a total of 30 subjects.

A total of 32 subjects were included in this study, meeting the minimum sample size requirement(Lenhard et al., 2017).

**Sensor Placement**(Skiba et al., 2022)**:**

| Opal sensors | Placement |
| --- | --- |
| Sternum | Centered on the flat surface of the chest, just below the collar bones |
| Lumbar | Centered at the base of the spine |
| Wrist * 2 | On the wrist, like a watch |
| Foot * 2 | Centered on the top of the foot, connector pointed towards the middle toe |

**The processing pipeline for MEPs data**(Rossi et al., 2021; S et al., 2023)**：**

For motor-evoked potentials (MEPs) calculation, surface electromyography (sEMG) was applied to record electrical potentials from the tibialis anterior (TA). The detailed parameters were as follows: a sampling rate of 5000 Hz, an amplifier set at 500, a notch filter at 50 Hz, and a low-pass filter at 500 Hz. Ten waveforms with good repeatability and representativeness were selected for analysis. MEP latency refers to the time taken for the motor response to occur after the stimulation of the motor cortex. It is calculated by measuring the time interval between the onset of stimulation and the onset of the MEP waveform. MEP amplitude refers to the magnitude or size of the electrical potential recorded from the muscles following stimulation. It is calculated by measuring the peak-to-peak amplitude, which is the difference between the peak and the trough of the MEP waveform.


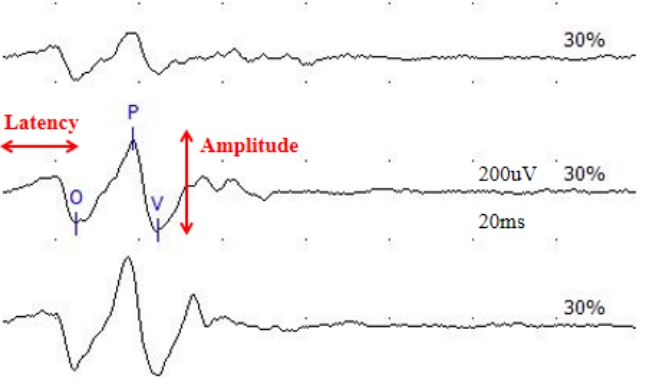


**Reference:**

Chieffo, R., Giatsidis, F., Santangelo, R., Alyagon, U., Comola, M., Zangen, A., Comi, G. and Leocani, L., Repetitive Transcranial Magnetic Stimulation With H-Coil Coupled With Cycling for Improving Lower Limb Motor Function After Stroke: An Exploratory Study, *Neuromodulation: Technology at the Neural Interface*, vol. **24**, no. 5, pp. 916–22, July 1, 2021. DOI: 10.1111/ner.13228

Lenhard, W. and Lenhard, A., *Computation of Effect Sizes*, 2017.

Rossi, S., Antal, A., Bestmann, S., Bikson, M., Brewer, C., Brockmöller, J., Carpenter, L. L., et al., Safety and Recommendations for TMS Use in Healthy Subjects and Patient Populations, with Updates on Training, Ethical and Regulatory Issues: Expert Guidelines, *Clinical Neurophysiology : Official Journal of the International Federation of Clinical Neurophysiology*, vol. **132**, no. 1, pp. 269–306, January 2021. DOI: 10.1016/j.clinph.2020.10.003

S, V., Kh, S. C., Mc, K., M, H., Dh, B., V, D. L., Pm, R., et al., Clinical Diagnostic Utility of Transcranial Magnetic Stimulation in Neurological Disorders. Updated Report of an IFCN Committee, *Clinical Neurophysiology : Official Journal of the International Federation of Clinical Neurophysiology*, vol. **150**, accessed March 11, 2024, from http://pubmed-ncbi-nlm-nih-gov-s.webvpn.njmu.edu.cn:8118/37068329/, June 2023. DOI: 10.1016/j.clinph.2023.03.010

Skiba, M. B., Harker, G., Guidarelli, C., El-Gohary, M., Horak, F., Roeland, E. J., Silbermann, R., Hayes-Lattin, B. and Winters-Stone, K., Using Wearable Inertial Sensors to Assess Mobility of Patients With Hematologic Cancer and Associations With Chemotherapy-Related Symptoms Before Autologous Hematopoietic Stem Cell Transplant: Cross-Sectional Study, *JMIR Cancer*, vol. **8**, no. 4, p. e39271, December 8, 2022. DOI: 10.2196/39271
